# PorB2/3 Protein Hybrid in *Neisseria meningitidis*

**DOI:** 10.3201/eid1404.070869

**Published:** 2008-04

**Authors:** Raquel Abad, Rocío Enríquez, Celia Salcedo, Julio A. Vázquez

**Affiliations:** * National Institute of Health Carlos III, Madrid, Spain

**Keywords:** PorB, meningococci, mosaic, letter

**To the Editor:** Class 2 and class 3 porin (PorB) proteins are the major proteins found in the outer membrane of *Neisseria meningitidis* (*1*); they function as porins, allowing the passage of small molecules through the outer membrane. PorB outer membrane proteins are transmembrane proteins with 8 predicted surface-exposed loops (I–VIII), which vary in length and in amino acid sequences. Several sequence analyses of these proteins have shown 4 regions with a high level of amino acid variability in loops I, V, VI, and VII (variable regions [VRs] 1–4) (*2*). The extensive antigenic variability of these proteins forms the basis of the *N. meningitidis* serotyping scheme (*3*,*4*). These 2 classes of proteins are mutually exclusive, and they are expressed by alternate alleles (*porB2* and *porB3*) at the *porB* locus (*1*).

All *N. meningitidis* strains received in the Spanish Reference Laboratory for *Neisseria* are routinely serotyped by whole-cell ELISA (*5*) with a set of monoclonal antibodies (MAbs) provided by the National Institute for Biological Standards and Control (South Mimms, UK) that includes the following serotypes: 1 (MN3C6B), 2a (5D4–5), 2b (MN2C3B), 4 (5DC4C8G8), 14 (MN5C8C), 15 (8B5–5G9), and 21 (6B11F2B5). Those meningococci that appear as nonserotypeable (NT) are analyzed by sequencing the *porB* gene (*6*). In the case discussed here, in the sequencing of a NT strain, the *porB* gene showed an unusual sequence.

This strain, isolated in Spain during 2006, was recovered from the cerebrospinal fluid of a patient with meningococcal disease. The *porB* gene sequence shows VR1–4, which is exclusive of PorB3 protein, and VR2-Eb, VR3–2ab, and VR4-Cc, which are typical of PorB2 (GenBank accession no. EF094023). A comparison of this new sequence with the available *porB* sequences in the *Neisseria*.org database (http://neisseria.org/nm/typing/porB) enabled a more detailed analysis of the fragments corresponding to *porB3* and *porB2* found in this sequence. The fragment from nt 1 to 213 was identical to the *porB3–193* allelic variant (VR1–4, VR2-Aa, VR3–7, VR4–14b), and the second part, with nt 233–972 identical to *porB2–99* (VR1-Dc, VR2-Eb, VR3–2ab, VR4-Cc). The region of 214–232 nt is identical in the 3 variants. Therefore, this is a true hybrid molecule, which appears to have arisen from recombinational events between *porB2–99* and *porB3–193* alleles. In fact, this finding has prompted the inclusion of a new family called porB2/3 hybrid in the *Neisseria.*org database to facilitate the collection of this type of *porB* sequences.

The most likely origin of the *porB2/3* hybrid (4, Eb, 2ab, Cc) is the acquisition of DNA that encodes a VR1–4 sequence by a meningococcus with a *porB2–99* allelic variant. It is less likely that DNA encoding the *porB* VR2-Eb, VR3–2ab, and VR4-Cc sequences was acquired by a meningococcus with the *porB3–193* allelic variant because a longer fragment of DNA would have been transferred.

In spite of the presence of a VR1–4, which should be recognized by the set of MAbs used, this strain appeared as NT. A Western blot assay using MAb type 4 showed a good recognition epitope-MAb. Therefore, the failure of MAbs to identify this strain may have been due to the limited accessibility of the epitope because of the alteration of the PorB protein, which might be affecting its conformation. Once again, genetic characterization should be a preferred method over phenotypic characterization for typing meningococcal strains. Molecular characterization of NT strains in other laboratories might clarify the true frequency of this event.

Intragenic recombination between porin genes of the same allelic family is likely occurring in nature because mosaic gene structure has been reported in *porB* genes. However, *porB2/3* recombinants have never been previously found in the nature. Given the known ability of meningococci to be transformed by DNA from other strains, it is surprising that occurrence of genuine *porB2/3* hybrids has not yet been documented. There is only a report of naturally occurring gonococci expressing a hybrid *porB1a*/*porB1b* (*7*) (PorB1a and PorB1b gonococcus porins, as in meningococci, are encoded by 2 families of diverged alleles of the *porB* gene [*8*]). Gonoccocal strains expressing the recombinant *por* genes appear to be particularly susceptible to the bactericidal effect of human serum (*9*). A similar situation might happen in *N. meningitidis*, with a selective disadvantage in the invasive process of these hybrid strains, explaining the rarity of naturally occurring hybrids. By contrast, mechanisms like this are frequently used by meningococci to avoid the immune response against ordinary antigens. The balance between advantages and disadvantages at this level would show the true implications of this event.

This finding is relevant regardless of its frequency in nature. This report suggests how frequent the recombination events should occur among the meningococcal population: even theoretical mutually exclusive genes can produce hybrid variants; such knowledge is an important step in the development of future vaccines based on protein formulations.
